# Sex-dependent association between coronary vessel dominance and cardiac syndrome X: a case-control study

**DOI:** 10.1186/1471-2261-14-142

**Published:** 2014-10-09

**Authors:** Zorin Makarovic, Sandra Makarovic, Ines Bilic-Curcic

**Affiliations:** Department of Cardiology, University Hospital Center Osijek and School of Medicine, University of Osijek, J. Huttlera 4, 31000 Osijek, Croatia; Department of Endocrinology, Diabetes and Metabolism Disorders, University Hospital Center Osijek and School of Medicine, University of Osijek, J. Huttlera 4, 31000 Osijek, Croatia

**Keywords:** Non obstructive coronary artery disease, Left coronary artery dominance, Mixed coronary artery dominance, Sex, Obstructive coronary artery disease

## Abstract

**Background:**

Previous studies have demonstrated the relevance of left coronary artery dominance in the outcome and prognosis of obstructive coronary artery disease (CAD). However, no studies have investigated the influence of coronary vessel dominance on non obstructive CAD. The aim of this study was to establish the association of left and mixed dominance of the major epicardial arteries with the development of non obstructive CAD and evaluate potential sex-dependent differences in the coronary artery supply.

**Methods:**

A total of 484 patients underwent the same diagnostic procedures. The patients were divided into two groups based on their coronary angiogram results: the control group (242 patients with obstructive CAD; coronary artery stenosis of ≥50%) and the experimental group (242 patients with non obstructive CAD; coronary artery stenosis of <50%).

**Results:**

Significantly more women than men were affected by non obstructive CAD (P = 0.005). Left dominance was more frequent in the non obstructive CAD group than in the control group (P = 0.018) and was more pronounced in women than in men (P = 0.013). Among men with non obstructive CAD, a left supply was more frequent than a mixed supply (P = 0.012). Women with non obstructive CAD had a higher frequency of a left supply, whereas a mixed supply was less frequent in men than in patients with obstructive CAD (P = 0.013 and 0.018, respectively).

**Conclusion:**

These results suggest that left dominance (particularly in women) and the absence of a mixed supply in men could cause regional ischemia, thus affecting the development of non obstructive CAD. Furthermore, sex may determine the incidence of specific coronary artery supply types, therefore influencing disease development and prognosis.

## Background

The concept of cardiac syndrome X (CSX) was introduced to clinical practice by Kemp et al. in 1973 to describe patients who developed angina during physical exercise with a normal coronary angiogram [1, 2]. Over time, this term began to encompass a broader spectrum of patients, including those with angina of any cause and the absence of significant coronary vessel changes [2–5]. Since 1967, when the first case of angina with normal coronarography findings was described, the definition of non obstructive coronary artery disease (CAD) has evolved from CSX and microvascular angina [6, 7] to microvascular coronary dysfunction [8].

Regardless of definitions and terminology, obstructive CAD indicates ≥50% stenosis of the coronary artery as shown on coronary angiography, while non obstructive CAD indicates <50% stenosis of the coronary artery [4]. These criteria are common to all definitions and understandings of this complex clinical syndrome.

The influence of sex on the development of non obstructive CAD has been demonstrated in previous studies. More than 50% of female patients evaluated for angina have non obstructive disease as shown by cardiac catheterization [9–11]. However, large epidemiological trials investigating possible risk factors are missing; specifically, there are currently no published data regarding the influence of the type of coronary artery supply in the development of non obstructive CAD.

Coronary artery dominance is determined by the artery that supplies the posterior descendent artery. Most individuals (around 85%) are right-dominant. Of the remaining patients, 8% are left-dominant and 7% are codominant [12–17]. Several studies have confirmed the relevance of left coronary artery dominance in the outcome and prognosis of obstructive CAD [18–20]. Therefore, it is conceivable that the type of coronary artery dominance also has an effect on the occurrence and outcome of non obstructive CAD.

The objective of this study was to establish the association of left dominance and codominance of major epicardial arteries with the development of non obstructive versus obstructive CAD and to evaluate potential sex-dependent differences in the coronary artery supply in non obstructive CAD.

## Methods

### Patients

This study complied with the Declaration of Helsinki and was approved by the ethics committee of University Hospital Center Osijek. The study participants were recruited from the population of patients who underwent coronary angiography for investigation of CAD at the Department of Cardiology, University Hospital Center Osijek. Written informed consent was obtained from each patient.

The inclusion criteria were as follows: age >18 years, left ventricle ejection fraction of ≥40%, angina, and a positive stress test (ergometry and/or myocardial perfusion scintigraphy). Although the clinical presentation of angina is usually typical, some patients with CAD present with atypical symptoms (up to 70% of patients according to some authors [21]); therefore, it was necessary to perform clinical tests that could accurately interpret the patients’ symptoms. All patients underwent noninvasive stress tests (ergometry or myocardial scintigraphy). A positive ergometry result was defined as significant changes of the ST segment (all five of the following criteria had to be met: horizontal or descendent ST-segment depression, ≥1-mm ST-segment depression, ≥80-ms duration of ST segment changes, ST-segment changes in at least two adjacent electric leads in the same ECG lead [22], and ST-segment changes in at least two adjacent ECG leads). Stress tests were considered positive if one of the following criteria was met: new onset of left bundle branch block (QRS duration of ≥120 ms) during a stress test, inversion of T waves during a stress test [23], or a >10-mmHg drop in blood pressure during a stress test.

Myocardial perfusion scintigraphy was performed in the following cases: patients with inconclusive stress test results, patients with negative stress test results, patients in whom significant clinical suspicion of CAD remained because of pronounced chest pain resembling angina during a stress test, patients with left bundle branch block who previously underwent a stress test, and patients already diagnosed with CAD if there was a need for additional evaluation of viable myocardium or residual ischemia (mainly cases in which the decision regarding myocardial revascularization was doubtful). Myocardial perfusion scintigraphy results were considered positive if reversible and/or irreversible defects of radionuclide uptake in the wall of the left ventricle occurred.

Patients with a history of the following conditions were excluded from the study: severe valvular heart disease, artificial heart valves, congenital heart disease, heart failure (left ventricle ejection fraction of <40%), hemodialysis with terminal renal insufficiency, contraindication to stress test and coronary angiography, immobility, advanced bilateral peripheral artery disease, permanent pacing, Wolff–Parkinson–White syndrome, previous percutaneous coronary intervention, previous coronary artery bypass grafting, pregnant, or breast-feeding.

All patients who met the inclusion criteria underwent routine coronary angiography based on the medical indications and guidelines of the European Society of Cardiology. Patients with coronary artery stenosis of <50% were diagnosed with non obstructive CAD, while patients with coronary artery stenosis of ≥50% were diagnosed with obstructive CAD. The dominance of the coronary artery supply was assessed during coronary angiography.

### Study design

This retrospective case-control study included 484 patients divided into two groups. The experimental group comprised 242 patients (143 women, 99 men) with established non obstructive CAD (coronary artery stenosis of <50% on coronary angiogram). The control group comprised 242 patients (141 women, 101 men) with obstructive CAD (coronary artery stenosis of ≥50% on coronary angiogram). The control group was sex matched with the experimental group (Table 1).

**Table 1:** **Sex distribution in the control and experimental groups**

Subjects	Experimental (non obstructive CAD) group (n)	Control (obstructive CAD) Group (n)	P value*
Females	143	141	0.888
Males	99	101	0.906

*Student’s t-test; CAD, coronary artery disease.

### Statistical analysis

Descriptive statistical analysis of the results was performed. Statistical significance was declared at a two-sided P value of 0.05 with 95% power unless otherwise specified. Statistical analyses were performed using SPSS software for Windows (version 16.0; SPSS Inc., Chicago, IL, USA).

Differences between the two independent variables were evaluated with the Student’s t-test for parametric analysis, while the chi-squared test was used for nonparametric analysis. Normal distributions of numerical variables were evaluated with the Kolmogorov–Smirnov test.

## Results

### Distribution of coronary artery dominance within the experimental group (non obstructive CAD) and control group (obstructive CAD)

The differences in the distribution of the dominant coronary artery supply between the two groups are summarized in Table 2. In the experimental group, more patients had left than mixed dominance, but the difference was not statistically significant (χ^2^ test, P = 0.075). In the control group, a significantly larger number of patients had mixed than left dominance (χ^2^ test, P = 0.024). As expected, a right coronary artery supply was dominant in both groups (74% in the non obstructive CAD vs. 77% in the obstructive CAD group).

**Table 2:** **Distribution of left and mixed coronary artery dominance within each group and between the experimental group (non obstructive CAD) and control group (obstructive CAD)**

Non obstructive CAD		Obstructive CAD		
Type of dominance	Frequency %	Type of dominance	Frequency %	P value*
Left	16	Left	8	0,018
Mixed	10	Mixed	15	0,096
P value*	0,075	P value	0,024	

*Chi-squared test; CAD, coronary artery disease.

Of the 242 patients with non obstructive CAD, 143 were women (59% of experimental group) and 99 were men (41%). This difference was statistically significant (χ^2^ test, P = 0.005).

### Frequency of left and mixed dominance in non obstructive versus obstructive CAD group

A left supply was observed at a significantly higher frequency in the non obstructive CAD group than in the control group (χ^2^ test, P = 0.018). In contrast, only a numerical difference in a mixed supply was present (χ^2^ test, P = 0.096) (Table 2).

### Distribution of coronary artery dominance in obstructive CAD according to sex

Sex-specific differences in the type of coronary supply in both groups are presented in Table 3. In the control group (obstructive CAD), dominance of a right coronary artery supply was present in the majority of men and women (72.5% vs. 77.3%, respectively) followed by a mixed supply (13.9% vs. 16.3%, respectively). Dominance of a left coronary artery supply was least frequent in both men and women (10.9% vs. 6.4%, respectively). These differences were not statistically significant with the exception of the mixed supply, dominance of which was significantly higher than the left supply in women (16.3% vs. 6.4%, respectively; χ^2^ test, P = 0.013).

**Table 3:** **Sex-specific distribution of coronary artery dominance in obstructive CAD versus non obstructive CAD group**

Gender	Group	Dominance	Frequency %	p value*
Males	Obstructive CAD	Left type	10,9	NS
		Mixed type	13,9	
	Non obstructive CAD	Left type	15,2	0,012
		Mixed type	4	
Females	Obstructive CAD	Left type	6,4	0,013
		Mixed type	16,3	
	Non obstructive CAD	Left type	16,8	NS
		Mixed type	14	
Males	Obstructive CAD	Mixed type	13,9	NS
Females			16,3	
Males	Non obstructive CAD	Mixed type	4	0,01
Females			14	

*Chi-squared test; CAD, coronary artery disease; NS, not significant.

### Distribution of coronary artery dominance in non obstructive CAD according to sex

In the experimental group (non obstructive CAD), women had a significantly higher frequency of a mixed supply than did men (14% vs. 4%, respectively; χ^2^ test, P = 0.01). The frequency of a left and right coronary artery supply was similar in women and men (16.8% vs. 15.2% and 69.2% vs. 80.8%, respectively) (Table 3). Among men with non obstructive CAD, a left supply was significantly more frequent than a mixed supply (χ^2^ test, P = 0.012), whereas in women, only a numerical difference existed and was not statistically significant (Table 3).

### Distribution of subjects according to coronary artery dominance and sex

Women with non obstructive CAD had a significantly higher frequency of left coronary artery dominance than did patients with obstructive CAD (χ^2^ test, P = 0.013). This numerical difference was also observed in men, but was not statistically significant (χ^2^ test, P = 0.433). Men with non obstructive CAD had a significantly lower frequency of mixed coronary artery dominance than did patients with obstructive CAD (χ^2^ test, P = 0.018), while in women, the difference was only numerical and not statistically significant (χ^2^ test, P = 0.647) (Table 4).

**Table 4:** **Distribution of patients according to type of coronary dominance and sex in obstructive CAD and non obstructive CAD groups**

Gender Subjects n = 484		Type of dominance		
		Left type (n)	Right type (n)	Mixed type (n)
Males	Obstructive CAD	11	76	14
	Non obstructive CAD	15	80	4
	P value*	NS	NS	0,018
Females	Obstructive CAD	9	109	23
	Non obstructive CAD	23	100	20
	P value*	0,013	NS	NS

*Chi-squared test; CAD, coronary artery disease; NS, not significant.

## Discussion

According to previous reports [12, 13], 85% of patients with CAD have a right coronary artery supply, 8% have a left supply, and 7% have a mixed (balanced) supply. These results were confirmed in studies investigating the implementation of coronary computed tomography angiography in establishing the prevalence of coronary artery variations and the effectiveness of this test in the diagnosis of coronary heart disease [14–17]. Our results confirmed the highest incidence of a right coronary artery supply in both groups, as expected. However, significantly more patients in the control group (obstructive CAD) had a mixed supply than a left supply. This discrepancy can be explained by the variations in the prevalence of specific types of supply in a precisely defined population; our patients were recruited from 2009 to 2012 from five eastern Croatian counties that comprise about 20% of the Croatian population. Additionally, the population selected for the present study originated from a pool of patients with a diagnosis of CAD and not from the general population; this differs from the populations described in previous studies.

Still, no reports in the literature have definitively confirmed the various distributions of different types of supply in the general population and in patients with obstructive CAD, although several studies have attempted to elucidate the importance of the coronary artery supply dominance in patients with obstructive CAD [18–20]. Veltman et al. [18] demonstrated that left coronary artery dominance is a predictor of nonfatal myocardial infarction and all-cause mortality in patients with CAD. Goldberg et al. [19] established that left coronary artery dominance is a significant and independent predictor of increased mortality in patients with acute coronary syndrome. In a study by Parikh et al. [20], most of the patients who underwent percutaneous coronary intervention because of acute coronary syndrome had left coronary artery dominance or a codominant supply and more frequently developed heart failure or cardiogenic shock. In our study, a mixed supply was present twice as frequently as in previous studies (15% vs. 7%, respectively), implicating a lower frequency of a right coronary artery dominance. Therefore, it is possible that our population might have been additionally compromised by adverse cardiovascular outcomes because of the increased incidence of a combined left and mixed supply. Because Indian Asians have a higher risk of cardiovascular death than do Europeans (which cannot be explained by either impaired microvascular circulation or other conventional risk factors [24]), we can speculate that other risk factors may be responsible, such as a genetically determined higher prevalence of left and mixed coronary artery dominance. Further prospective studies are needed to investigate and compare disease outcomes between our study population and populations from other areas of Croatia, Europe, or the rest of the world to confirm this assumption.

A higher frequency of a mixed coronary artery supply than a left coronary artery supply was observed in women with obstructive CAD. This can be explained by the fact that the remaining women with a left supply were actually present in the non obstructive CAD group. Indeed, a significantly higher number of women with left dominance were present in the non obstructive than the obstructive CAD group.

In contrast to the control group, left coronary artery dominance occurred twice as often in patients with non obstructive CAD as in previously published data (16% vs. 8%, respectively), implying that the dominant type of supply to the myocardium has a role in the development of non obstructive CAD. In such cases, the right coronary artery would be less developed then the circumflex coronary artery, which could cause regional ischemia in the area supplied by the right coronary artery regardless of the presence of normal coronary arteries. This notion is supported by the observation that women in the non obstructive CAD group had a higher frequency of a left supply than did women in the obstructive CAD group. However, the same finding was not observed in men, implying that this difference could be sex specific or that a larger number of patients are needed to prove statistical significance in men.

Men with non obstructive CAD had a significantly lower frequency of a mixed supply than a left supply, indicating that dominance of a mixed coronary artery supply could be less frequent in patients with non obstructive CAD. Additionally, significantly more women than men in the non obstructive CAD group had a mixed supply. These results favor the assumption that the absence of dominance of a mixed supply, at least in men, is associated with an increased occurrence of regional ischemia because the myocardium is equally supplied by the right coronary artery and circumflex coronary artery. Admittedly, those observations were confirmed only in men, not in women, which again suggests the possibility of a sex-specific difference or the need to further increase the sample size to obtain statistical significance for women.

Finally, significantly more women than men were affected by non obstructive CAD, consistent with previous reports [9–11].

The present study has several limitations. The patient population was relatively small and recruited exclusively from an east region of Croatia. This may have resulted in a different prevalence of coronary vessel dominance than in previous reports [12–17]. The small sample could also explain the different findings between the men and women in both groups. Moreover, because this was a case-control study, disease outcome and prognosis in relation to specific types of coronary vessel dominance remain unknown. Thus, a large-scale long-term prospective study is needed to confirm the present results and determine the relationship between coronary vessel dominance and the prognosis of non obstructive CAD.

## Conclusions

In summary, among patients with non obstructive CAD, a left coronary artery supply was more frequent in women, possibly affecting regional ischemia in the area of the right coronary artery. A mixed coronary artery supply was less frequent in men, probably resulting in an increased occurrence of regional ischemia owing to the lack of a balanced supply present in the mixed type. These findings may indicate a possible role of sex in the evolution of non obstructive CAD.
